# microRNAs trip down memory lane

**DOI:** 10.15252/emmm.202114997

**Published:** 2021-10-20

**Authors:** Nancy S Yacovzada, Eran Hornstein

**Affiliations:** ^1^ Department of Molecular Genetics Weizmann Institute of Science Rehovot Israel; ^2^ Department of Molecular Neuroscience Weizmann Institute of Science Rehovot Israel

**Keywords:** Biomarkers, Neuroscience, RNA Biology

## Abstract

A new study by Islam *et al,* in this issue of *EMBO Molecular Medicine,* reports three microRNAs in the blood that are linked to inter‐individual differences in cognition, prior to any sign of cognitive decline. The novel miRNA biomarkers may assist in predicting the risk of cognitive decline and later of developing dementia and can contribute to decision strategies about early therapeutic interventions.

## Graphical abstract

N. Yacovzada and E. Hornstein discuss the study by Islam *et al* (in this issue of *EMBO Mol Med*) that reports three circulating miRNAs that are linked to inter‐individual differences in cognition and may assist in predicting the risk of cognitive decline.

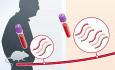

The prevalence of cognitive decline and dementia in the human population is estimated to double every two decades, imposing a growing challenge to society. Mild cognitive impairment (MCI, or mild neurocognitive disorder) is an acquired deficit in cognitive ability that is typically progressive, affecting skills such as learning and problem‐solving, memory, and perception. Individuals diagnosed with MCI have an annual risk of ˜ 10% of conversion to dementia, especially to Alzheimer’s disease (AD, Fig 1A).

**Figure 1:**
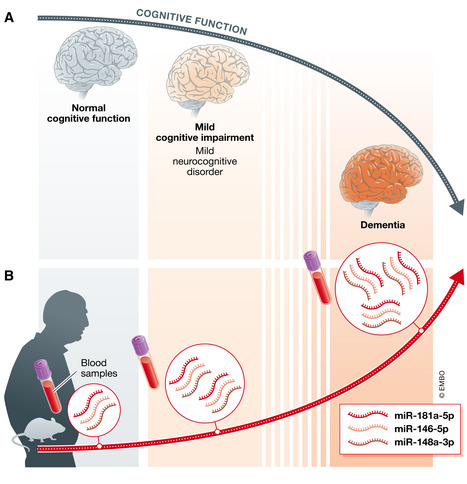
A three miRNA signature of impaired cognition in mice and human blood (A) In some older individuals, normal cognitive function is prone to deteriorate into mild cognitive impairment (known also as mild neurocognitive disorder), which is considered a prodromal state of dementia, including primarily Alzheimer’s disease. (B) In a new work by Islam *et al* (2021), authors study human blood samples and mouse models of aging and dementia. They demonstrate a reciprocal correlation between brain function and the levels of three endogenous microRNAs in the blood, namely, miR‐181a‐5p, miR‐146‐5p, and miR‐148a‐3p. Intriguingly, the blood levels of these three miRNA increase when brain neurocognitive capacity deteriorates. The discovery of the three miRNA signature holds promise for potential future development of tests that will contribute to clinical judgment of the risk or degree of cognitive impairment.

Minimally invasive laboratory tests that assist the diagnosis of cognitive status are an unmet need. Although brain imaging or measuring the levels of proteins, such as amyloid beta or tau, in the blood or cerebrospinal fluid (CSF) is considered minimally invasive, today's tests are proven useful only in the advanced stage of dementia (Beach, 2017; Molinuevo *et al*, 2018). This limits their utility because late in the disease, treatment options are extremely limited. Therefore, it is an important challenge to discover new markers that can be utilized for screening the seemingly healthy population for the earliest signs of cognitive decline.

In this issue of *EMBO Molecular Medicine*, Islam *et al* (2021) propose an original approach for screening healthy individuals at risk of cognitive decline. The approach is based on blood drawing and hence is “minimally invasive” and features the quantification of microRNAs (miRNAs) in the blood.

miRNAs are endogenous small RNAs that post‐transcriptionally regulate gene expression. miRNAs play crucial roles in brain maintenance and are essential for neuron survival (Schratt, 2009). miRNAs have been implicated in the pathogenesis of neurodegeneration and can contribute to biomarker development for cognitive impairment and dementia including AD (Cogswell *et al*, 2008; Kayano *et al*, 2016; Barbagallo *et al*, 2020; Dong *et al*, 2020; Wei *et al*, 2020; Zhao *et al*, 2020).

The underlying reasons for using miRNAs as biomarkers is that small RNAs are present in tissues, including blood and brain cells, but are also detectable outside cells. Their expression can inform about tissue and disease status. The mechanism by which miRNAs exit cells is still under extensive research. However, two main working hypotheses stand out: miRNAs may be spilled out of dying cells or may be secreted in a regulated manner in extracellular vesicles. Equally important for biomarker development is the fact that miRNAs can be quantified in a high throughput manner by modern RNA sequencing techniques. Therefore, relatively rapid analysis of hundreds of miRNA species in large cohorts of human samples opened up in recent years a new field of biomarker discovery.

Utilizing such a framework, Islam *et al* (2021) addressed an intriguing research mission: to identify miRNAs that are informative about the cognitive state in healthy humans. They performed their analysis by correlating miRNA signatures in blood to the score gained by cognitive assessment tests.

The authors further proceeded in an original way to combine data gained from humans with a longitudinal analysis of miRNAs in aging mice. This integrative approach, which combines two very different mammal species, enabled prioritization and cross‐validation. This certainly increases confidence in the three miRNA hits, namely, miR‐181a‐5p, miR‐146a‐5p, and miR‐148a‐3p (Fig 1B). Furthermore, the authors demonstrated that these three miRNAs together predict cognitive decline in an independent human cohort. Finally, Islam *et al* (2021) use mice to provide initial proof that the three miRNAs may not only inform about changes in cognitive status but also might contribute to neural homeostasis and may be potentially involved in the molecular mechanism of cognitive decline.

The strategy of Islam *et al* (2021) prioritizes miRNAs with similar measured patterns across two species. Therefore, miRNAs measured in blood became relevant to the study only if their signal overcame differences associated with comparing human cognition and its mouse counterpart. Accordingly, the same miRNA molecules must be detected in association with a sophisticated neuropsychological assessment in humans and with one key memory function in mice.

Such an integrative approach is valuable because phenomena conserved across species are more likely to be genuine. However, the authors take into account that they overlook human‐specific changes and miRNA genes that are present in the *Homo sapiens* genome but not conserved to the mouse genome. Together, room is given for future research to explore human‐specific features and to incorporate longitudinal human data to directly test actual changes in the course of time.

In the same work, the authors went on to further demonstrate that inhibition (knockdown) of the three miRNAs with “anti‐microRNA” synthetic oligonucleotides, can therapeutically mitigate cognitive impairment in aging mice and in a mouse model of Alzheimer’s disease. This part of the work is impactful twofold: First, the anti‐miRNA molecules reported, may be developed in the future, into new experimental drugs for cognitive decline and dementia therapy. Of course, follow‐up studies will be required to understand how anti‐miRNAs work in this context and their pharmacology. Second, the fact that manipulating the levels of the three miRNAs ameliorates deterioration also implicates them in molecular axes of cognitive decline and suggests that the increased levels in the blood are directly, perhaps mechanistically, linked to congestive decline. However, a better understanding of potential causality will require additional studies in animal models and longitudinal studies in human cohorts.

A couple of notable challenges will be addressed in the future by experts in this emerging field: As the analysis of Islam *et al* (2021) is primarily based on whole blood RNA, where a substantial amount of the RNA is from blood cells, it is currently unknown if the expression of the three microRNAs originated from brain, other organs, or alterations in the composition of white or red bloods cells. Measurements of the miRNAs in cerebrospinal fluid might suggest a potential brain origin, and re‐analysis of another study’s data implies that the miRNAs could be detected in the blood's liquid portion. In addition, microRNAs in the blood may be derived from extracellular vesicles of the brain origin (Hill, 2019). Therefore, additional research will probably contribute to a better understanding of the miRNAs' tissue sources and the molecular mechanism that dictates changes in the miRNA levels in the blood.

Finally, major challenges lay ahead toward future point‐of‐care utilization of biomarkers, including miRNA biomarkers. Simple means for measurements and standardization of blood miRNA levels in the clinic should be developed. Furthermore, the computational development of a robust personalized predictor that can forecast an individual’s risk, based on absolute miRNA values, will also be needed.
